# Thermoelectric Measuring Equipment for Perioperative Monitoring of Temperature and Heat Flux Density of the Human Eye in Vitreoretinal Surgery

**DOI:** 10.3390/s25040999

**Published:** 2025-02-07

**Authors:** Roman Kobylianskyi, Krzysztof Przystupa, Valentyn Lysko, Jacek Majewski, Lyudmyla Vikhor, Vadym Boichuk, Oleg Zadorozhnyy, Orest Kochan, Mykola Umanets, Nataliya Pasyechnikova

**Affiliations:** 1 Institute of Thermoelectricity of the NAS and MES of Ukraine, 58029 Chernivtsi, Ukraine; romakobylianskyi@ukr.net (R.K.); v.lysko@chnu.edu.ua (V.L.); vikhorl@ukr.net (L.V.); boichuk.vadym.vi@chnu.edu.ua (V.B.); zadoroleg2@gmail.com (O.Z.); 2 Department of Thermoelectricity and Medical Physics, Yuriy Fedkovych Chernivtsi National University, 58012 Chernivtsi, Ukraine; 3 Department of Automation, Lublin University of Technology, Nadbystrzycka 38D, 20-618 Lublin, Poland; 4 Institute of Mechanical Science, Vilnius Gediminas Technical University, J. Basanavičiaus Str. 28, LT-03224 Vilnius, Lithuania; 5 Department of Automatics and Metrology, Lublin University of Technology, Nadbystrzycka 38D, 20-618 Lublin, Poland; j.majewski@pollub.pl; 6 State Institution “The Filatov Institute of Eye Diseases and Tissue Therapy of the National Academy of Medical Sciences of Ukraine”, 65015 Odesa, Ukraine; filatovretina@gmail.com (M.U.); pasnv2017@gmail.com (N.P.); 7 Department of Measuring Information Technologies, Institute of Computer Technologies, Automation and Metrology, Lviv Polytechnic National University, Stepana Bandery Str. 12, 79013 Lviv, Ukraine; 8 Department of Specialized Computer Systems, Faculty of Computer Information Technologies, West Ukrainian National University, Lvivska Str. 11, 46009 Ternopil, Ukraine

**Keywords:** thermoelectric device, temperature, heat flux, vitreoretinal surgery, proliferative diabetic retinopathy

## Abstract

Perioperative monitoring of the ocular heat transfer is important for increasing the safety of long-term vitreoretinal surgery. The study is aimed at studying new thermoelectric measuring devices for comprehensive perioperative monitoring of ocular temperature and heat fluxes in vitreoretinal surgery. This pilot, open-label, prospective study included 23 patients (23 eyes) with proliferative diabetic retinopathy (PDR) in both eyes. The thermoelectric devices were developed for measuring intraocular temperature in vitreoretinal surgery and for determining the ocular surface temperature (OST) and heat flux (HF) density. In all cases, OST and HF density of both eyes were recorded (before and after surgery). Intraocular temperature and temperature of the irrigation fluid were measured intraoperatively. No complications associated with the perioperative use of thermoelectric temperature and HF sensors were identified during the study. The successful application of thermoelectric temperature and HF sensors, developed specifically for ophthalmological applications, in comprehensive perioperative monitoring of ocular heat transfer in patients with PDR in vitreoretinal surgery was demonstrated for the first time. Further research is needed to confirm the benefits of perioperative temperature monitoring in vitreoretinal surgery, as well as to develop equipment for active management of temperature in surgical practice.

## 1. Introduction

Measuring body temperature for diagnostic purposes has been known since ancient times. As early as in the 5th century BC, Hippocrates used the difference between hot and cold to identify certain diseases. Quantitative assessment of body temperature became possible in early 1600, when Galileo developed the first thermometer [1]. The earliest attempts to measure ocular surface temperature (OST) using a mercury thermometer were made in the 19th century [2]. The OST can now be assessed using contact thermometry with temperature sensors that require direct contact with the cornea or conjunctiva. Temperature sensors can even be a component of a “smart” contact lens [3,4]. Moreover, OST can be assessed contactless by recording thermal infrared (IR) radiation, namely, by infrared (IR) thermometry or thermography methods [5].

Contact thermometry methods, unlike contactless, allow for the study of both ocular surface and intraocular temperatures. However, intraocular thermometry is only advisable during surgical intervention due to the existing incisions for instruments, for example, to monitor the temperature of intraocular contents during vitreoretinal surgery [6].

Today, vitreoretinal surgery is the “gold” standard for treating patients with severe ophthalmological pathology, including proliferative diabetic retinopathy, penetrating eye injuries, and retinal detachment. Despite the constant improvement of surgical technologies, a number of unresolved problems remain that reduce the success of surgical treatment [7]. Standard vitreoretinal surgery often neglects ocular heat transfer during and after the procedure. Irrigation fluid temperature, intraocular temperature, and ocular surface temperature are typically not monitored, and ophthalmic measurement equipment is unavailable. Thus, it was previously established that standard vitreoretinal surgery is accompanied by artificial uncontrolled deep hypothermia of intraocular structures with their subsequent rapid warming, which can be dangerous for retinal cells. This is due to the use, for certain stages of vitreoretinal surgery, of irrigation fluid at room temperature, i.e., significantly lower than the temperature of the intraocular media [6,7]. It should be noted that vitreoretinal surgical interventions are often lengthy (120 min or more) [8], and there are neither clear standardized recommendations on temperature conditions for surgery that are safe for the retina, nor instructions on the safe rate of recovery of intraocular temperature, nor the appropriateness of monitoring eye temperature in the postoperative period.

The development of inflammatory complications in the postoperative period is also an important problem in intraocular surgery [9]. Currently, only the laser photometry method is an objective method that allows assessing the presence and determining the degree of intraocular inflammation [10]. However, its widespread use is limited by the need for complex and expensive equipment. The search for means of objectively assessing the presence of intraocular inflammation, including subclinical inflammation, using accessible and reliable methods remains an urgent task in ophthalmology. It is known that inflammation of the eye tissues, which can be a dangerous complication of surgery and requires urgent measures, is often accompanied by hyperthermia of the eye [11]. To date, significant progress has been made in the creation of means for measuring temperature and heat flux (HF), which is an important indicator of heat exchange, and together with thermometry allows deeper understanding of the functional state of organs and tissues, including the eye tissue [12,13]. Modern thermoelectric HF sensors combine compactness, high sensitivity, accuracy, and speed [14,15]. Therefore, recording changes in temperature and HF due to inflammation looks promising and can be used for early detection and quantification of postoperative intraocular inflammation.

Thus, intraoperative monitoring of intraocular temperature and perioperative monitoring of the thermal characteristics of the eye are important conditions for increasing the safety of long-term vitreoretinal surgery [16,17]. To solve the abovementioned problems in the field of vitreoretinal surgery and to gain a comprehensive understanding of the thermal processes occurring in the ocular tissues during surgery and in the postoperative period, the development and implementation of high-precision medical equipment for measuring temperature and heat fluxes for ophthalmological needs are relevant.

Therefore, the aim of this study was to explore the possibility of using new thermoelectric measuring devices for comprehensive perioperative monitoring of ocular temperature and heat fluxes in vitreoretinal surgery.

## 2. Materials and Methods

At the Institute of Thermoelectricity of the National Academy of Sciences (NAS) and the Ministry of Education and Science (MES) of Ukraine, together with the State Institution “The Filatov Institute of Eye Diseases and Tissue Therapy of the National Academy of Medical Sciences of Ukraine” (FIEDTT) within the framework of a cooperation agreement, the thermoelectric device for measuring intraocular temperature during vitreoretinal surgery [18] was developed. There was also developed another thermoelectric device for determining the temperature and HF on the ocular surface [19,20].

### 2.1. Thermoelectric Device for Measuring Intraocular Temperature

**Figure 1:**
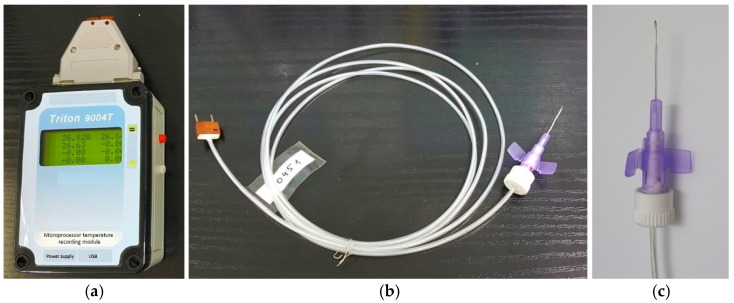
Appearance of the thermoelectric device for measuring intraocular temperature. (**a**) microprocessor temperature recording module; (**b**) flexible thermocouple measuring probe; (**c**) measuring probe in a standard polytetrafluoroethylene cannula housing (the outer diameter of the measuring probe is 0.6 mm; its length is 19 mm).

The multichannel thermoelectric device consists of a microprocessor temperature recording module, thermocouple measuring microprobes, a docking device, and a computer with software for real-time recording and visualizing of temperature readings, shown in (Figure 1).

The temperature measurements are transferred to a computer by a USB cable. The technical characteristics of the thermoelectric device are given in Table 1.

Microprobe temperature sensors are based on the type-L thermocouple (iron-constantan). The thermocouple sensor is placed inside of a standard polytetrafluoroethylene cannula (outer diameter of the measuring probe 0.6 mm, length 19 mm). The diameter of the working part of the probe allows it to be used intraoperatively through a standard port for vitrectomy. The thermocouple junction is welded to a heat concentrator made of medical stainless steel and fixed to the end of the cannula. The thermocouple wires pass from the cannula into a 1.5-meter-long cable and end with a plug. The tightness of the measuring probe is ensured by the use of medical silicone sealant, which is chemically neutral and allows for thermal or chemical sterilisation (like a conventional medical instrument).

Microprobes are connected to the microprocessor temperature recording module via a docking device using a plug. The docking module has 4 sockets, to which up to 4 microprobes can be connected simultaneously. The temperature recording module is connected to the docking device by DB-37f connector (KLS Electronic, Ningbo, China).

**Figure 2:**
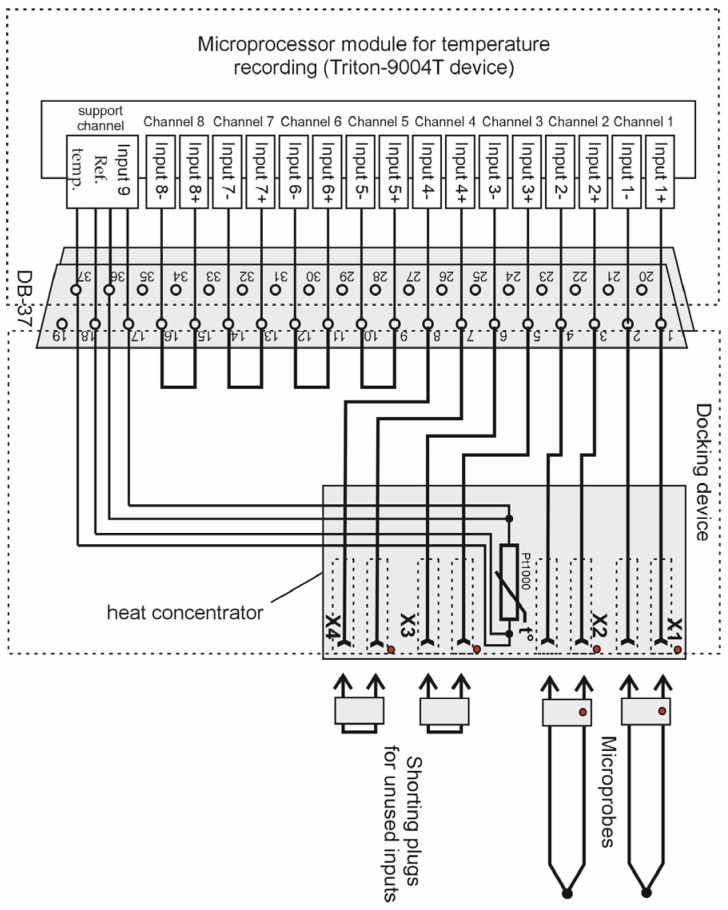
Diagram of connecting thermocouple microprobes to the developed thermoelectric device for measuring intraocular temperature.

The sockets in the docking device are mounted on a copper heat concentrator, which also houses a precision temperature sensor (the platinum resistance thermometer PT-100). It measures the temperature of the reference junctions of the thermocouples, i.e., the reference temperature. The connection diagram of the microprobes to the microprocessor temperature meter is shown in Figure 2.

The microprocessor module for temperature recording is based on Triton-9004T device, which has an 8-channel 24-bit analog-to-digital converter. This temperature meter uses 4 channels, and the other 4 channels can be used additionally. The maximum input voltage of the measuring channel is ±1.17 V. The temperature recording module can be powered either by a rechargeable battery or by a network adapter. Using such an adapter, the rechargeable battery is charged. The battery can also be recharged and the device can be operated from a computer via a USB cable.

The ThermoLogger v.2.0 program (Terex, Kyiv, Ukraine) was used to work with the multichannel temperature measurement module. It allows for real-time measurements and reading of data from the memory block. The measurement results are displayed on the screen using a graph and a table, which can be saved, exported, and printed.

The developed device provides the ability to set the sensitivity separately for each channel of the microprocessor module with respect to the chosen type of thermocouples. The thermoelectric device can measure the temperature during a preset time interval in the range of 4 s to 2 h. The measurements are recorded in non-volatile memory. The memory capacity of the device is 50 thousand cells. The programming of the microprocessor module channels is performed using a computer.

### 2.2. Ensuring High Accuracy of the Thermoelectric Device for Measuring Intraocular Temperature

**Figure 3:**
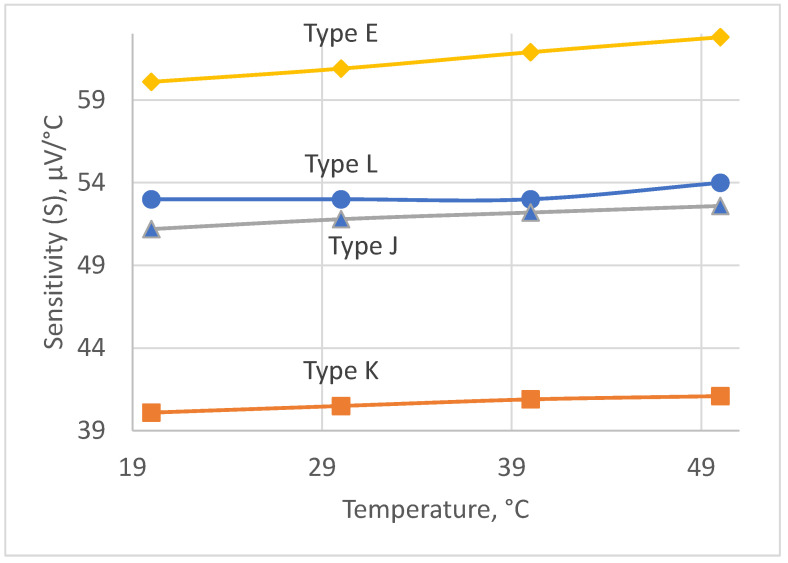
Sensitivity of thermocouples.

The choice of the thermocouple is governed by the following requirements: (i) the measurement range is 20–50 °C; (ii) a high sensitivity in the chosen range; and (iii) a high stability of its sensitivity. The four types of thermocouples that fit best these requirements are as follows: type E, type L, type K, and type J [21,22]. The plots of sensitivities versus measured temperature for the abovementioned types of thermocouples in the range of 20–50 °C are given in Figure 3.

The most suitable type is type L, because of its quite high sensitivity and best stability of its sensitivity within the chosen temperature range. As can be seen, its sensitivity is constant within the range of 20–40 °C and equals 53 μV/°C for the temperatures above 40 °C. Its sensitivity gradually changes from 53 μV/°C to 54 μV/°C within the range of 40 °C to 50 °C. However, we expect that measurements in this subrange will occur rarely, because we aimed at detecting the inflammation earlier, below 40 °C, and in the early 40 °C in the worst case to minimize the measurement error.

The thermocouple has many merits, such as immunity to vibrations and robustness to mechanical stresses. The thermocouple has problems of degradation of their legs under the influence of operation temperature and time. It leads to the appearance of two interconnected errors due to drift [23,24] and inhomogeneity [25,26,27]. The latter has been known since 1906 [28] but still remains a big problem [29], which attracts the considerable attention of researchers [30,31,32,33]. However, both errors appear because of the degradation of thermocouple legs during operation under the influence of operating temperature and time [25]. The rate of degradation can be roughly estimated using the Arrhenius equation [34]

(1) $$k=Ae^{-E_{a}/RT}$$

where *k* is the rate constant, *e* is the base of natural logarithms, *T* is the absolute temperature, *R* is the universal gas constant, *E_a_* is activation energy, and *A* is the frequency factor. This equation contains two unknown variables, *A* and *E_a_.* Let us determine the ratio of rates of chemical reactions at two different temperatures *T*_1_ and *T*_2_

(2) $$\frac{k_{2}}{k_{1}}=e^{-\frac{E_{a}}{R}\left(\frac{1}{T_{2}}-\frac{1}{T_{1}}\right)}$$

Let us now substitute the numerical values as follows: *R* = 8314 J⋅K^−1^⋅mol^−1^, *T*_2_ = 1023 K and *T*_1_ = 323 K, which correspond to 750 °C and 50 °C, respectively. The latter figure is used because in our case we measure temperatures in the vicinity of 0 °C. The type L thermocouple consists of iron (positive) and konstantan (negative) legs [35]. The order of activation energy can be estimated from [36]. For the sake of convenience of calculations, we assume *E_a_* = 41 kcal, or *E_a_* = 171,544 J. For this case, the ratio of rates equals approximately 9 × 10^18^. Unfortunately, we have not found the data about error due to inhomogeneity for type L thermocouples, but there are data for type J thermocouples [32], the composition of which is similar. The maximum inhomogeneity is about 600 μV, or 11.5 °C at the temperature of operation of 750 °C. Thus, for the operation temperature of 50 °C, we can expect inhomogeneity to be 9 × 10^18^ times less, which is negligibly small.

Error due to drift can be estimated from data given in [37]. For the type J thermocouple, it is negligible. That is why we can expect the conversion characteristics of type J thermocouples to be stable in the chosen temperature range. The only considerable error in this case is tolerance. According to [22], the tolerance is either ±1.5 °C or ±3 °C, with respect to the accuracy class.

When the intraocular temperature for the same patient is measured within several days using the same instrument and sensor, then it can be referred to as relative measurements [38]. In this case, all systematic errors cancel out, and only the instability during these several days matters. As mentioned above, the instability of the sensor is negligible in the chosen temperature range.

The accuracy of the sensor can be further improved by calibrating it before the measurements. The procedure of calibration is as follows. The thermocouple is placed similarly to the medical thermometer together with it to measure the actual axillary body temperature. In this case, the error of reproduction of the temperature fixed point of the human body does not exceed 0.2 °C [39]. It should be noted that the measured temperature is going to be within ±5 °C from the axillary temperature. Thus, the calibrated type L thermocouple can also be used for absolute measurements of intraocular temperature with the adequate accuracy.

### 2.3. Thermoelectric Device for Measuring the Temperature and HF Density on the Ocular Surface

**Figure 4:**
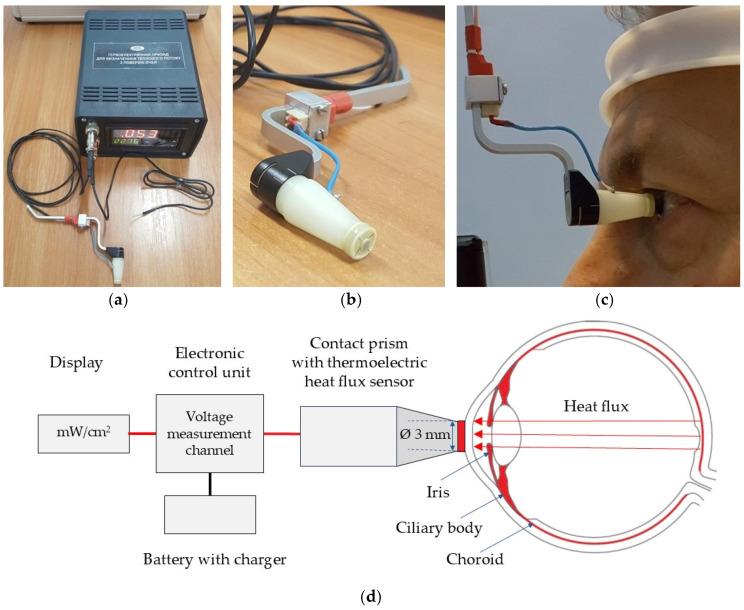
The thermoelectric device for determining the temperature and HF density of the human eye [15]. (**a**) Electronic control unit; (**b**) Thermoelectric HF sensor, located between two ceramic plates (3 mm in diameter); (**c**) The device attached to a slit lamp; (**d**) Schematic illustration of the device’s design and operation principle for measuring the HF density from the ocular surface.

A multichannel thermoelectric device for determining the density of HF from the ocular surface is designed as an autonomous device with a battery power source. This allows for conducting highly accurate measurements of heat fluxes and temperatures of biological objects by the contact methods. The appearance of such a device is shown in Figure 4.

The thermoelectric HF sensor is located between two ceramic plates. It is fixed to a specially made contact prism and installed in a standard mount for contact prisms of the Goldmann applanation tonometer. The position of the contact prism with the HF sensor with respect to the patient’s eye during operation is given in Figure 4c. The multi-channel device for determining the HF density of the eye consists of an electronic control unit and the thermoelectric HF sensor. For the specified thermoelectric device, a miniature thermoelectric HF sensor was developed and manufactured using a special patented technology of the Institute of Thermoelectricity of the NAS and the MES of Ukraine. The thermoelectric micromodule with the dimensions of (2 × 2 × 0.5) mm contains 100 semiconductor crystals of n- and p-type of conductivity with the dimensions of (0.17 × 0.17 × 0.4) mm of a highly efficient thermoelectric material based on Bi-Te. Such a thermoelectric micromodule is placed between two ceramic plates based on Al_2_O_3_ with a diameter of 3 mm and a thickness of 0.1 mm each, and the side surface is sealed using a special sealant. Thus, the diameter and height of the manufactured thermoelectric HF sensor are 3 mm and 0.7 mm, respectively.

The HF sensor is mounted on a specially manufactured contact prism. The prism with the HF sensor is installed in a standard mount for contact prisms of the Goldmann applanation tonometer and can be connected to slit lamps of various manufacturers. In our study, the thermoelectric device for determining the HF density from the ocular surface was adapted to a slit lamp (Carl Zeiss SL-130, Jena, Germany) equipped with the Goldmann applanation tonometer (Carl Zeiss AT 020, Jena, Germany).

A feature of the design of the contact prism is that it can be removed for disinfection after each inspection of each patient. The thermoelectric HF sensor, which is fixed in the centre of the contact prism, is in direct contact with the outer surface of the human eye (in our study with the centre of the cornea).

The contact surface of the thermoelectric HF sensor is made atraumatic (with smoothed edges). The possibility of disinfection of this surface is provided. The thermoelectric HF sensor (the diameter of 3 mm) is located in the center of the working surface of the contact prism (the diameter of 7 mm) and a small optical control zone between them is structurally provided so that a doctor, looking through the biomicroscope, can accurately install the specified thermoelectric sensor on the centre of the cornea.

In the electronic unit, the device has its HF measurement channel, which is designed to accurately measure the generated voltage of the HF thermoelectric sensor and its subsequent conversion into a physical quantity in units of HF density (mW/cm^2^). The resolution of the voltage measurement channel is ±1 μV, which allows HF measurements to be made with the maximum accuracy. The device also has a temperature measurement channel, which is designed for high-precision temperature measurement by a thermocouple, as well as an ambient temperature measurement channel. A chromel–copel thermocouple is used as a temperature sensor, which can be manufactured with minimal geometric dimensions. This allows for measuring the temperature of miniature biological objects at high speed. The technical characteristics of the device are given in Table 2.

The digital microcontroller is designed to control measurement channels, and to normalize and convert generated signals into physical quantities. The digital microcontroller can be programmed using the buttons located on the front panel of the device, selecting the sensor type and measurement limits.

The battery power supply with a charger is designed for galvanic isolation of the device and the biological object being studied to prevent electric shock. Due to the device’s galvanic isolation from the power grid, safe and effective use of the device in ophthalmological practice is ensured. The low voltage of the autonomous power supply of the device (no more than 4.5 V) does not pose a threat of electric shock to any biological object being studied. This also virtually eliminates normal mode noise and thus considerably improves the accuracy of measurements of both heat flux and temperature.

The digital display shows the measurement results (the HF density in mW/cm^2^ and the temperature in °C) on the front panel of the device. The LED digital display is large and bright, which allows for carrying out measurements in darkened rooms from long distances.

The device is simple, compact, portable, autonomous, and reliable in operation, allowing medical staff to use it without special training. Therefore, the technical advantages of such a device include the presence of the highly sensitive specific thermoelectric HF sensor, the ability to measure temperature with the sensitivity of ±0.01 °C, the safe of use of the device due to its galvanic isolation from the power grid, and the ability to monitor the thermal and temperature state of the surface of the human eye in real time.

The calibration of the thermoelectric HF sensor was carried out and the coefficient for converting the value of the generated voltage of the thermoelectric sensor into a physical quantity in units of HF density (mW/cm^2^) was determined.

### 2.4. Calibrating the HF Measurement Channel

**Figure 5:**
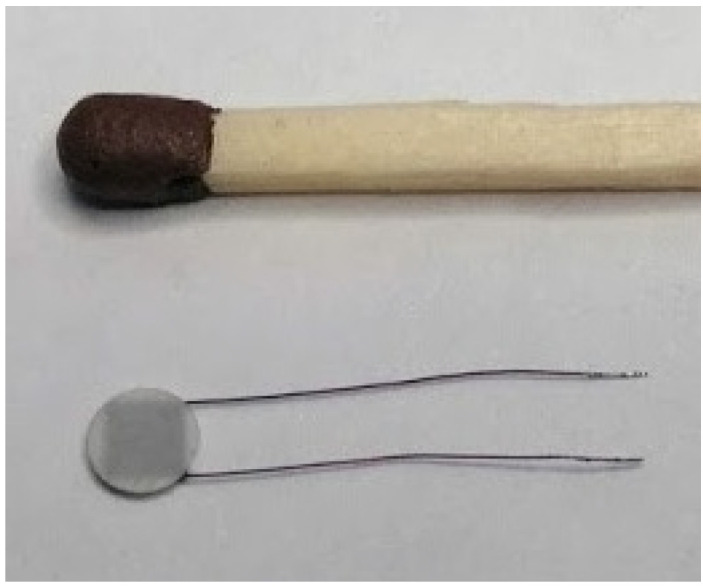
The designed thermoelectric heat flux sensor [14,15].

**Figure 6:**
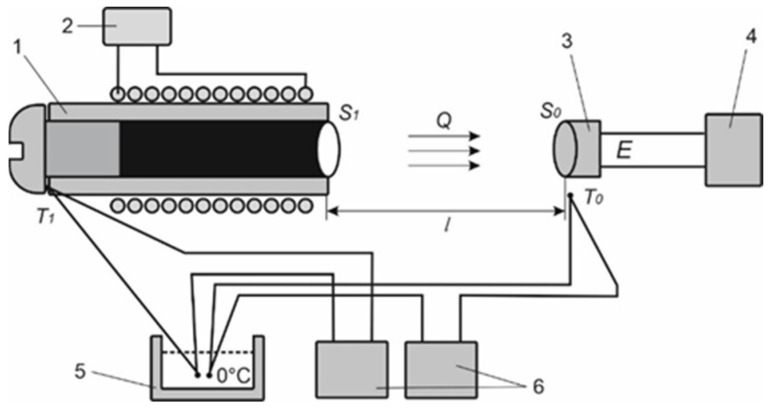
The apparatus for calibrating the heat flux sensor to determine its volt–watt sensitivity [14,15]: 1—blackbody, 2—power supply unit of the blackbody heater, 3—thermoelectric heat flux sensor, 4—millivoltmeter, 5—zero-thermostat of the thermocouple, 6—temperature meters.

The developed HF sensor [14,15] (see Figure 5) needs calibration before use. The calibration apparatus for determining the volt–watt sensitivity [14,15] is based on the blackbody radiator as the heat flux source. The apparatus is given in Figure 6.

The volt–watt sensitivity of the developed sensor is determined from the formula

(3) $$\nu =\frac{E}{Q}$$

where *E* is thermoEMF developed by the sensor and *Q* is the heat flux radiated by the black body and absorbed by the thermoelectric sensor. The heat flux *Q* is determined from the formula below:

(4) $$Q=\frac{\varepsilon_{1}\varepsilon_{2}\sigma \left(T_{1}^{4}-T_{0}^{4}\right)S_{1}S_{0}}{\pi l^{2}}$$

where σ is the Stefan–Boltzmann constant, ε_1_ = 1 for the black body radiator, ε_2_ = 0.82 for the receiving pad of the sensor, *T*_1_ is the temperature of the blackbody package, *T*_0_ is the temperature of the receiving pad, *S*_1_ is the area of radiating hole of the blackbody, *S*_0_ is the area of the receiving pad, and *l* is the distance between the hole and the receiving pad.

After the calibration of the HF sensor, the conversion factor *K* = 1/ν*S*_0_ for the sensor to the physical quantity in units of heat flux density is determined. The value of this conversion factor for the developed sensor is determined to be *K* = 4.163 mW/mV·cm^2^ [14].

When the heat flux for the same patient is measured within several days using the same instrument and sensor, then it can be referred to as relative measurements [14,38]. In this case all systematic errors cancel out and only the instability during the measurement series matter. As mentioned above, the instability of the sensor is negligible in the chosen temperature range. This instability can be considered as no greater than 1%.

### 2.5. Patients

All procedures performed in this pilot, open-label, prospective human study met the ethical standards outlined in the Declaration of Helsinki and the relevant laws of Ukraine. Approval from the Bioethics Committee of the State Institution “FIEDTT” was obtained before the study (14 May 2018; approval number: 6). All individual participants were required to provide a written consent statement to participate in the study before any procedures related to the study were performed.

This study included 23 patients aged from 37 to 55 years (23 eyes) with proliferative diabetic retinopathy (PDR) in both eyes.

Inclusion criteria were patients with type 2 diabetes, and the presence of PDR in both eyes.

Exclusion criteria were the presence of acute or chronic intraocular or periocular inflammatory processes, the presence of neovascular glaucoma, and any previous intraocular surgery.

In all cases, the patient’s body temperature, the temperature, and the HF density on the corneal surface of both eyes were recorded (one day before the surgery and on the 3rd day of the postoperative period). The intraocular temperature in the vitreous cavity, the temperature of the irrigation solution supplied to the eye, and room air temperature were measured intraoperatively. Laser photometry of the aqueous humour of both eyes was performed (one day before the surgery and on the 3rd day after the surgery) using the FM-600 device (Kowa Co., Nagoya, Japan).

Patients with PDR in both eyes underwent standard three-port pars plana vitrectomy in one eye. Vitrectomy was performed using the Constellation Vision System (Alcon Laboratories, Inc., Fort Worth, TX, USA).

### 2.6. Technique for Measuring the Temperature and HF Density on the Ocular Surface and Intraoperative Measurement of Intraocular Temperature

Before the start of pre- and postoperative measurements of HF density and the temperature of the outer corneal surface, patients spent 15 min indoors to adapt to the environment. The next step in all cases was epibulbar anaesthesia of both eyes in the form of a single instillation of 0.5% proxymetacaine hydrochloride solution (Alcaine; SA Alcon-Couvreur NV, Puurs, Belgium) at room temperature. Thermal measurements were performed 15 min after the application of the drops. During the study, the subject was in a sitting position behind a slit lamp. The sensor of the thermoelectric device in all cases was in contact with the central area of the cornea. At least three measurements of each eye were performed in real time. The study was conducted in a room with stable environmental conditions and at a specific time of day (between 15:00 and 16:00). The conditions were maintained with minimal air velocity in the room. The study was conducted without drug-induced pupil dilation.

#### Intraoperative Thermometry

After creating surgical access and installing standard 23 G instrument ports, a flexible thermocouple measuring probe of a thermoelectric device was inserted into the vitreous cavity through the port. Intraoperative temperature measurement was performed in real time in the middle part of the vitreous cavity of the eye (adhering to the conditional anterior–posterior axial line of the eye) and at least three measurements were performed at each stage of the study. The initial temperature in the vitreous body was recorded before vitrectomy; the temperature of the vitreous contents immediately after vitrectomy, accompanied by a continuous irrigation process; and after completion of all subsequent stages of surgery (endolaser coagulation of the retina, removal of the internal limiting membrane of the retina, removal of the epiretinal membrane, straightening of the retina with perfluorodecalin). The duration of each stage of vitreoretinal surgery was also recorded. BSS PLUS balanced salt solution (Alcon Laboratories, Inc., Fort Worth, TX, USA) at room temperature was used for vitrectomy. The temperature of the irrigation fluid entering the eye and the ambient temperature in the operating room were measured. The temperature of the irrigation solution was monitored during the surgery. After the surgery, the patients were observed for 7 days.

### 2.7. Statistical Analysis

The data were expressed as the mean (M) ± the standard deviation (SD). The normality of data distribution was tested using the Shapiro–Wilk test [40]. A paired Student’s *t*-test [41] was used to compare HF density/OST in paired eyes, the HF density/OST before and after surgery, and the intraocular temperature before and after vitrectomy. Statistical significance was set at *p* < 0.05. Statistical analysis was performed using Statistica software (Version 10.0, StatSoft, Tulsa, OK, USA). GraphPad Prism 8.0 software (Version 8.0.1, GraphPad Software, San Diego, CA, USA) was used to create figures.

## 3. Results

The mean age of patients (13 men, 10 women) with type 2 diabetes and bilateral PDR in the presented study was 46 ± 6 years. The room temperature during the measurements of the HF density and the OST was on average 22.5 ± 0.7 °C.

**Figure 7:**
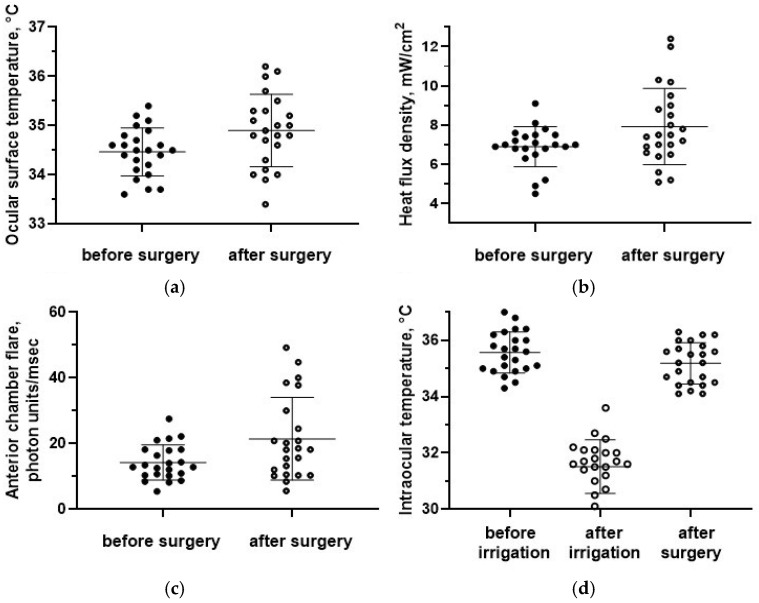
(**a**) The ocular surface temperature, (**b**) the HF density, and (**c**) anterior chamber flare before and after surgery. (**d**) The intraocular temperature dynamics. Data are expressed as mean ± standard deviations (M ± SD).

The preoperative OST was equal to 34.5 ± 0.5 °C. It was not significantly different from the paired eyes (34.6 ± 0.5 °C; *p* = 0.4). The mean postoperative OST (34.9 ± 0.7 °C) was significantly higher compared with the preoperative values (*p* = 0.02, Figure 7a) and became significantly different compared with non-operated eyes (*p* = 0.02).

The baseline HF density on the ocular surface was equal to 6.9 ± 1.0 mW/cm^2^ and was also not significantly different from the paired eyes, 7.2 ± 1.1 mW/cm^2^ (*p* = 0.4). The HF density after surgery (7.9 ± 1.9 mW/cm^2^) was significantly higher compared with the baseline values (*p* = 0.03, Figure 7b) and compared to intact fellow eyes (*p* = 0.02).

Laser photometry values after surgery (21.4 ± 12.5 f/ms) were also significantly higher compared with the preoperative period (13.7 ± 5.1 f/ms; *p* = 0.009, Figure 7c).

The air temperature in the operating room before the start of vitreoretinal surgery was equal to 24.8 ± 0.5 °C, the temperature of the irrigation solution was equal to 24.5 ± 0.4 °C, and the patient’s body temperature was equal to 36.5 ± 0.3 °C.

The initial vitreous temperature was equal to 35.6 ± 0.7 °C, which was significantly higher than the preoperative OST (34.5 ± 0.5 °C; *p* = 0.00). The intraocular temperature immediately after vitrectomy (31.6 ± 0.9 °C) was significantly lower than the initial values (*p* = 0.00, Figure 7d). However, after additional manipulations during surgery, the intraocular temperature rapidly increased to 35.2 ± 0.7 °C and did not differ from the initial values (*p* = 0.08, Figure 7d). The rate of recovery of the vitreous temperature was equal to 0.3 ± 0.1 °C/min.

In four cases, both an increase in the OST of the operated eye by more than 1.0 °C compared to the initial values and an asymmetry of more than 1.0 °C between the OST values of the paired eyes were noted in the postoperative period. In addition, in these cases, an increase in the HF density of the operated eye was recorded compared to the initial measurements. These patients also had the highest values of laser photometry (>30 ph/ms) in the operated eyes. In two eyes, clinical signs of postoperative inflammation were observed (moderate light scattering in the aqueous humour of the anterior chamber of the eye, cells, and cellular aggregates).

In addition, for one patient, in the postoperative period, asymmetry of the HF density values between the paired eyes was determined, with a significant increase in the HF density of the eye compared to the initial values (from 7.4 mW/cm^2^ to 12.4 mW/cm^2^) with minor temperature changes, which was accompanied by an increase in laser photometry values (up to 40 ph/ms) and clinical signs of intraocular inflammation.

No complications associated with the perioperative use of thermoelectric temperature and HF sensors were identified during this study.

## 4. Discussion

This study demonstrated the possibility of using thermoelectric devices for comprehensive perioperative monitoring of ocular temperature and heat fluxes during vitreoretinal surgery in patients with PDR.

Thus, at the first stage of the work, using a slit lamp-adapted thermoelectric device for recording HF of the eye, the HF density and OST of patients in the preoperative period was determined. First of all, we assessed the symmetry of the measurements between the right and left eyes. We did not register a significant difference in the HF density and OST of paired eyes. This observation is obviously due to the fact that patients with the same stage of diabetic retinopathy in both eyes participated in the study. It is known that heat exchange indicators in patients with different stages of diabetic retinal damage may differ due to changes in the choroid, which is the main source of heat in the human eye [13,42].

In the postoperative period, we observed an increase in the average values of OST and HF density of the eye, as well as asymmetry in the thermal characteristics of paired eyes, which indicates changes in the heat transfer of the operated eye. An increase in laser photometry indicators was recorded, which characterizes the state of the blood–ocular barrier and is an objective indicator of intraocular inflammation [43]. It has previously been reported that some patients after cataract surgery, glaucoma surgery, and vitreoretinal surgery had subclinical inflammation confirmed by laser photometry [43,44,45]. We assume that the changes in the temperature and HF density on the ocular surface, recorded in the postoperative period, may be a consequence of postoperative inflammation (mostly subclinical) and the concomitant intensification of blood circulation in the choroid of the eye [7,11,46]. The role of inflammation in changes in ocular heat exchange is also confirmed by the clinical picture in some patients. Thus, in three cases, clinical signs of inflammation were detected; increased laser photometry values were determined, caused by changes in the blood–ocular barrier due to the inflammatory process, as well as increased HF density values on the surface of the operated eye and the OST. The absence of clinical signs of infectious complications in these patients may indicate the aseptic nature of the inflammatory response of the eye tissues to surgical trauma [47].

Previously proposed optical methods for quantitative assessment of intraocular inflammation are influenced by several factors that affect the values of light scattering intensity in the anterior chamber: corneal transparency, the presence of cataract or intraocular lens, and a shallow anterior chamber [48,49,50]. These factors have a reduced impact on ocular heat transfer. Together with a simpler examination technique and less expensive equipment, the proposed method demonstrates the potential of HF density measurements for objectively assessing postoperative ocular inflammation.

At the stage of intraoperative studying of intraocular temperature, the thermoelectric device equipped with flexible thermocouple measuring probes was used, which was successfully applied during surgery through standard ports for surgical instruments.

We utilized thermoelectric measuring probes made from a material with low thermal conductivity, which enhances measurement accuracy by minimizing heat loss. It is known that temperature measurements of ocular tissues obtained using metal probes can be several degrees lower than those measured by probes with low thermal conductivity [51]. The duration of temperature measurement at each stage of the operation was 15–20 s. No complications resulting from the use of the measuring instrument during the surgery were registered. Thus, thermoelectric sensors provide safe intraoperative monitoring of intraocular temperature in vitreoretinal surgery. In addition, the developed device provided reliable monitoring of the temperature of the irrigation fluid during all stages of the surgical intervention. This represents a significant advancement in the practical use of targeted temperature management in vitreoretinal surgery.

Our study shows that standard vitreoretinal surgery with room temperature irrigation fluid is performed under conditions of uncontrolled hypothermia of the eye followed by rapid uncontrolled warming of the vitreous cavity after the cooling stage, which is consistent with reports from other authors [6,52,53]. Thus, at an irrigation fluid temperature of 24.5 °C, the intraocular temperature difference after vitrectomy was 4 °C, and the temperature recovery occurred very quickly (0.3 °C/min). Such rapid changes in intraocular temperatures pose a risk of damage to the retina due to cooling, as well as the occurrence of undesirable vascular reactions during the operation [16,17,54,55].

The main drawbacks of the thermoelectric sensors in this study is that they must be in contact with ocular tissues to carry out the measurements. This increases the risk of damage to both the ocular surface and the intraocular structures. Simultaneously, the proposed devices offer high accuracy, a wide temperature measurement range, the capability for point measurements, simplicity of design, and low cost.

Our study has the following limitations: (1) there is studied a relatively small sample size; (2) we did not focus on analysing the relationship between ocular heat transfer and treatment outcomes; (3) it does not allow us to compare the effectiveness of different temperature modes for surgery in the treatment of patients with PDR, as well as to determine the optimal modes for vitreoretinal surgery, which will be the subject of our further research. This pilot study primarily focused on the feasibility of using the proposed thermoelectric devices for comprehensive perioperative monitoring of ocular thermal processes. Further research and long-term outcomes will clarify the benefits and potential issues of the developed thermoelectric equipment for temperature monitoring in vitreoretinal surgery and the objective early diagnosis of postoperative inflammation. Increasing the sample size in subsequent studies may improve the generalizability and reliability of the findings.

Despite some limitations, the results showed that the medical thermoelectric equipment used to measure intraocular temperature, epibulbar temperature, and HF density was safe and effective for monitoring the thermal processes of the eye throughout all stages of vitreoretinal surgery. For the first time, the possibility of comprehensive perioperative monitoring of ocular thermal processes in patients with PDR was demonstrated using thermoelectric temperature and HF sensors developed specially for ophthalmological applications. Our study provides the initial findings on objectively assessing postoperative intraocular inflammation through the ocular HF density measurement. To enhance the practical application of research findings, it is beneficial to create a model for identifying and predicting postoperative intraocular inflammation across various types of pathologies and surgical procedures using multi-task and multimodal approaches [56,57].

We believe that to increase the effectiveness and safety of vitreoretinal surgery, it is advisable to introduce a perioperative temperature management system, which provides the following: (1) intraoperative monitoring and management of the temperature of the irrigation fluid and intraocular temperature to prevent hypothermia of the intraocular structures; (2) monitoring and management of the rate of rewarming of the vitreous cavity to avoid dangerous vascular reactions during surgery; and (3) monitoring the OST and the ocular surface HF density in the postoperative period for early detection and objective monitoring of intraocular inflammation.

## 5. Conclusions

Thermoelectric sensors that measure temperature and HF density on the ocular surface in patients with PDR after vitreoretinal surgery allow for detecting intraocular inflammation, even at a subclinical level. In addition, they allow for its quantitative assessment.

The thermoelectric device for intraocular thermometry in patients with PDR during vitreoretinal surgery allows for monitoring the temperature of the irrigation fluid, the level of intraoperative hypothermia, and the rate of recovery of intraocular temperature.

Confirmation of the benefits of comprehensive perioperative temperature monitoring in vitreoretinal surgery and implementation of this technique in surgical practice requires further targeted research and development of equipment, not only for monitoring but also for management of the temperature of irrigation solutions and intraocular temperature.

## Figures and Tables

**Table 1:** Technical characteristics of the thermoelectric device for measuring intraocular temperature.

Technical Characteristics	Value
Temperature measurement range	(−10 ÷ +120) °C
Temperature measurement error	±0.08 °C
Number of temperature measurement channels	4
Temperature recording period	from 4 s to 2 h
Diameter of microprobes for temperature measurement	0.6 mm
Real-time temperature measurement	possible
Continuous operation time of the device from fully charged batteries	100 h
Device power supply:	
Li-Ion battery 950 mA/h	+
network adapter AC220 V/DC12 V, 1 A	+
Charging batteries via USB interface	+
PC data exchange interface type	USB
Overall dimensions of the microprocessor temperature recording module	(125 × 90 × 60) mm
Overall dimensions of the docking device	(70 × 55 × 25) mm
Device weight	0.5 kg

**Table 2:** Technical characteristics of the device for measuring the temperature and HF density on the ocular surface.

Technical Characteristics of the Device	Parameter Values
Number of measurement channels	4
Number of thermoelectric HF sensors	1
Number of thermocouples	1
HF density measurement range	0.01 ÷ 50 mW/cm^2^
HF density measurement error	±5%
Temperature measurement range	0 ÷ 50 °C
Temperature measurement sensitivity	0.01 °C
Room temperature measurement range	0 ÷ 50 °C
Room temperature measurement resolution	0.01 °C
Battery voltage measurement range	3.7 ÷ 4.5 V
Continuous operation time of the device from a charged battery	100 h
Overall dimensions of the thermoelectric HF sensor	Ø3 × 0.7 mm
Overall dimensions of the electronic control unit	180 × 140 × 90 mm
Weight	0.6 kg

## Data Availability

Data are contained within the article.
